# Supplementary material for: Treatment of Winery Wastewater by Combined Almond Skin Coagulant and Sulfate Radicals: Assessment of HSO5− Activators
Source: Int J Environ Res Public Health. 2023 Jan 30;20(3):2486. doi: 10.3390/ijerph20032486 (PMC9916210; doi:10.3390/ijerph20032486)
Supplement: Supplementary file 1 [file ijerph-20-02486-s001.zip › ijerph-2163210-supplementary.pdf]

International Journal of
Environmental Research and Public Health

Supplementary material

Treatment of Winery Wastewater by Combined Almond Skin Coagulant and Sulfate Radicals: Assessment of HSO_5^- Activators

Nuno Jorge [1,2] *, Ana R. Teixeira [2], Lisete Fernandes [2], Sílvia Afonso [3], Ivo Oliveira [3], Berta Gonçalves [3], Marco S. Lucas [2] and José A. Peres [2]

1 Escuela Internacional de Doctorado (EIDO), Campus da Auga, Campus Universitário de Ourense, Universidade de Vigo, As Lagoas 32004, Ourense, Spain
2 Centro de Química de Vila Real (CQVR), Departamento de Química, Universidade de Trás-os-Montes e Alto Douro (UTAD), Quinta de Prados 5000-801, Vila Real, Portugal
3 Centre for the Research and Technology of Agro-Environmental and Biological Sciences—CITAB, University of Trás-os-Montes e Alto Douro, UTAD, Quinta de Prados, 5000-801 Vila Real, Portugal
* Correspondence: jperes@utad.pt (J.A.P.)

Table S1. Concentration of total phenolic, flavonoids, O-diphenol and DPPH present in almond skin extract.

	Total phenolic	Flavonoids	O-diphenol	DPPH
	mg GAE/g	mg CE/g	mg CAE/g	µg Trolox/g
ASE	6.51 ± 0.62	4.30 ± 0.16	0.65 ± 0.05	8.93 ± 0.34

Table S2. Analysis of variance.

Source	DF	Adj SS	Adj MS	F-Value	P-Value
Model	9	4308.97	478.77	6.43	0.027
Linear	3	3419.70	1139.90	15.30	0.006
PMS	1	2204.48	2204.48	29.59	0.003
Co^{2+}	1	846.66	846.66	11.36	0.020
Radiation	1	368.56	368.56	4.95	0.077
Square	3	432.91	144.30	1.94	0.242
PMS*PMS	1	229.71	229.71	3.08	0.139
Co^{2+}*Co^{2+}	1	219.63	219.63	2.95	0.147
Radiation*Radiation	1	36.93	36.93	0.50	0.513
2-Way Interaction	3	456.35	152.12	2.04	0.227
PMS*Co^{2+}	1	408.04	408.04	5.48	0.066
PMS*Radiation	1	0.01	0.01	0.00	0.991
Co^{2+}*Radiation	1	48.30	48.30	0.65	0.457
Error	5	372.52	74.50		
Lack-of-Fit	3	372.52	124.17	*	*
Pure Error	2	0.00	0.00		
Total	14	4681.49			

Table S3. Model summary.

**Standard deviation (SD)**	**R-squared**	**Adj R-squared**	**Predicted R-squared**
8.63160	92.04%	77.72%	0.00%
